# The Effect of Musical Stimulation and Mother’s Voice on the Early Development of Musical Abilities: A Neuropsychological Perspective

**DOI:** 10.3390/ijerph18168467

**Published:** 2021-08-11

**Authors:** Ilona Poćwierz-Marciniak, Michał Harciarek

**Affiliations:** Institute of Psychology, University of Gdansk, 80-309 Gdansk, Poland; michal.harciarek@ug.edu.pl

**Keywords:** early musical development, mother’s voice, musical stimulation, musical development, neuropsychology of music, neuroplasticity

## Abstract

An infant’s early contact with music affects its future development in a broad sense, including the development of musical aptitude. Contact with the mother’s voice, both prenatally and after birth, is also extremely important for creating an emotional bond between the infant and the mother. This article discusses the role that auditory experience—both typically musical and that associated with the mother’s voice—plays in fetal, neonatal, and infant development, particularly in terms of musical aptitude. Attempts have also been made to elucidate the neuropsychological mechanisms underlying the positive effects that appropriate musical stimulation can have on a child’s development.

## 1. Introduction

Children navigate the world of sound and acquire their first auditory experiences as early as in prenatal life. This develops their auditory sensitivity and memory, resulting in a readiness to process auditory information, which lays the foundation for later development of musical aptitude [1] as well as for language learning [2]. However, especially after birth, the interaction of genetic factors with diverse acoustic stimulation intensifies the development of individual musical aptitude [3]. In addition, listening to the mother’s voice has a great impact on building a bond with her [2,4,5]. Furthermore, musical development, taking place in the presence of musical stimulation, shows certain regularities during different periods of life. In this context, how do auditory experiences affect the fetus, and how do they affect the newborn and infant? In this article, we have reviewed a broad range of research reports that help to answer this question. Neuropsychological mechanisms explaining the role that early auditory stimulation plays in child development, particularly musical development, have also been discussed.

## 2. Prenatal Period

Musical development in fetal life occurs through the interaction of genetically determined auditory sensory development with the incoming auditory stimulation received by the fetus [3,6,7,8]. In the case of the sense of hearing, the developing ear follicles are visible as early as at 6 weeks of fetal life [9]. In the 10th week, the inner ear develops intensively—hair cells form on the basal membrane in the cochlea [1], so that, between the 14th and 15th week, the organ of Corti, although not yet mature, starts its functioning. As early as roughly the 25th week of pregnancy, the developing auditory system reacts to sounds, which is manifested, among other things, by accelerated heart rate and increased fetal mobility [10,11]. Between weeks 24 and 28, both the auditory receptors themselves (hair cells) and their synaptic connections mature. Between the 26th and 28th week, myelination of the auditory pathways occurs, affecting the speed and synchronization of nerve impulse flow. Initially—from roughly week 25 to week 27—the auditory system responds to a low frequency range of 250–500 Hz; later—from weeks 29 to 31—it expands to 1000–3000 Hz. As the ability to respond to a wider range of frequencies increases, the ability to discriminate between individual frequencies develops, the sensitivity to perceive quiet sounds increases, and the temporal processing associated with the increasingly rapid perception of sounds, including those of short duration, develops [1]. Fetal auditory sensitivity, understood as the ability to process very quiet sounds, matures particularly intensively between weeks 24 and 35 [12]. Loud music results in accelerated heart rate and increased motor activity, whereas quiet music has the opposite effect [13]. It is worth noting that fetal ears are filled with amniotic fluid; therefore, the sense of hearing is protected from damage that could be caused by excessively loud sounds [14].

By the third trimester of pregnancy, the sense of hearing already functions similarly to that of an adult [15]. For example, measuring the maturity of the response to music (e.g., Brahms’ Lullaby)—by assessing heart rate and motor responses—has shown that a significant change in auditory processing occurs around week 33 [16]. Around the time of birth, infants not yet born present very precise changes in their physiological responses, such as changes in heart rate or motility, depending on specific aspects of musical stimulation (e.g., intensity, frequency, timbre) [17,18,19,20] and changes in tempo [21]. Further, studies using magnetoencephalography (MEG) have also provided evidence for a developing ability to discriminate changes in intensity and pitch during the third trimester. For example, Rossitz Draganov et al. [22] used single, appropriately modulated tones without music as stimulation. In their study, a continuous acceleration of the brain’s response to changes in sound frequency has been shown to occur between the 27th week of pregnancy and birth [22]. Additionally, it has been demonstrated that between the 31st and 40th week, the brain’s response to changes in sounds occurs as early as at the very beginning of a sound’s duration [23]. Hence, as also posited by other researchers, such results demonstrate the significant maturity of the auditory system during the third trimester of pregnancy [23,24].

Functional magnetic resonance imaging (fMRI) findings also provide interesting data. Indeed, it has been proven that, from the 33rd week, there is a distinct response of the primary auditory cortex in the left hemisphere to single tones (Heschl’s area) [25]. This allows us to conclude that, as early as in the beginning of the third trimester, sound processing goes beyond the reflex level determined by the subcortical system [24]. Further, a recent fMRI study has demonstrated that, a few days before delivery, a temporal and frontal activation in response to guitar music may be seen [26]. Moreover, it has also been shown that the voice of a singing mother may activate the temporal lobe of the fetus [27].

These research results clearly indicate that certain musical aptitude develops as early as in fetal life, especially auditory sensitivity to changes in pitch and intensity and to changes in tempo, which, in terms of musical development, is reflected in musical hearing. The main process responsible for the development of this sensitivity is the maturation of the central nervous system, determined by genetic factors and supported by the presence of auditory impressions originating from the external environment. Auditory stimulation affects the branching of neuronal connections and their maintenance, increasing the readiness to process auditory information after birth.

Undoubtedly, musical memory also constitutes an important musical aptitude. Does it then also appear already in fetal life? Research conducted over many years proves that this is indeed the case. It is worth noting that the primary sound world surrounding the not-yet-born child is associated with the mother’s body. The fetus hears the noise of flowing blood, intestinal and gastric activity, heartbeat and breathing, as well as the sound of the mother’s steps and, most importantly, her voice [13], perceived at a volume 8 dB louder than that of other persons [28]. It appears that, after birth, newborns recognize their mothers’ voices and they can distinguish them from other women [29,30]. In addition, presenting newborns with the sounds of the human body (heartbeat, breathing) results in calmness, providing a sense of security [2]. This suggests that sound memory is already present in the fetus during prenatal life. Sounds, including music, which the baby was frequently exposed to during fetal life, are clearly recognized by the baby after birth. This is supported by the results of Peter Hepper’s experiment [31], in which children in the fetal period regularly heard the musical theme of an American television series. At birth, they responded to this music fragment with calmness, a lowered pulse rate, and decreased mobility. The repetition of stimuli influences recall and increases the sense of security from the prenatal period.

Another example of learning as early as in the womb is the phenomenon of auditory habituation, observed in the third trimester of pregnancy [11,32,33]. It involves adapting to repeated loud or unexpected sounds from the external environment. The first motor and physiological responses of the fetus are indicative of a strong stress response, whereas after repeated exposure to the same stimuli, this response is slowed down. This effect is interpreted as a memory that the sounds in question are not threatening. For example, evidence supporting this notion comes from studies of newborns whose mothers moved near the airport while still pregnant. Specifically, it has been demonstrated that the loud sound of an airplane passing by was more likely to wake and cause crying in those babies whose mothers lived near the airport only in the last weeks of pregnancy. In comparison, children whose mothers already lived in the vicinity of the airport during the first half of their pregnancy somehow became accustomed to the sound of flying planes [10].

Overall, research results indicate that the more often in the prenatal period the child heard a particular sound or music, the stronger the memory of these stimuli was after the birth [1,34,35]. However, the permanence of memory traces from fetal life is debatable. Some investigators speculate that such traces may persist for up to a year [36]. Support for this postulate comes from one neurophysiological study, in which a simple lullaby was presented from a recording a few times a week, starting from the 29th week of pregnancy until birth. Measurement of the event-related potentials at birth and four months later showed that children who listened to a lullaby had a significantly stronger brain response compared to children who had not received such stimulation during the fetal period. Moreover, the effect was related to the intensity of stimulation; the more times the lullaby was listened to in fetal life, the greater was the brain response to these sounds after birth [34]. Undoubtedly, the development of learning processes in the prenatal period is conditioned by the interaction of the maturing nervous system with repeated stimulation, which allows the emergence of the long-term synaptic reinforcement responsible for the consolidation of memory traces.

Musicianship seems to be of great importance for musical development. It is understood as the ability to experience music emotionally, to discover the emotional message while listening, and to express emotions naturally while performing music [37]. When analyzing reports about the role that maternal singing plays both in fetal life and after birth, one can venture to say that musicality is formed already in the prenatal period, largely owing to maternal singing. What is the mechanism accounting for this effect? The amniotic fluid surrounding the fetus is an effective transmitter of the melodic contour of the maternal voice. When a mother speaks or sings, the prosodic properties of her voice, such as melody and rhythm, are transmitted to the developing child through sound waves that travel through the tissues of her body. At the same time, the mother’s emotional state, reflected in the tone of voice, its timbre and frequency, and in the rhythm and tempo of speaking or singing, is associated with specific hormonal activity. This specific hormonal code that accompanies the emotions experienced by the mother is passed along with the blood to the child, inducing specific neurotransmitter activity. Thus, the perception of emotion becomes associated with the specific melodic and rhythmic features of the mother’s voice, especially during her singing. As a result, the interwoven dual-channel auditory–emotional experience even before birth forms the basis of the ability to perceive and understand emotions in both speech and singing, and later also in music [5,38].

According to researchers, the sensitivity to the prosodic properties of the maternal voice and language, which is formed already in fetal life, provides evidence not only for the ability of the fetus to perceive sounds, but also for its ability to attend to, process, and memorize simple auditory information [39]. An infant’s musical and language abilities begin to form even before birth. Of note, it should be kept in mind that heredity interacts with the environment. Thus, the contact of the fetus with music is a prerequisite for musical development as early as in the prenatal period [2,3], while full symbiosis with the mother guarantees contact with her voice and native language. It is during pregnancy that the mother introduces her yet unborn child to the world of sounds, music, and the songs she sings, expanding the child’s ability to develop musical hearing, a sense of rhythm, musical memory, and musicality.

## 3. Neonatal Period (0–1st Month of Life)

The child comes into the world with a developed sense of hearing, allowing the perception of music. A healthy newborn can differentiate the pitch, timbre, and intensity of sounds and has the aptitude to temporally process musical stimuli (duration, rhythm, and tempo), all of which, as mentioned earlier, were already developing in the third trimester of pregnancy [20]. However, it is worth noting that despite the maturity of the sense of hearing, the perception of music does not yet take place in the same way as in an adult. This is because the newborn does not yet have the aptitude to understand complex musical structures [1]. Listening to music cannot be limited to processing simple acoustic stimuli. It is a process involving cognitive (e.g., complex auditory perception, attention, or memory), emotional, and motivational functions. In addition, one cannot forget about the entire system of associations, expectations, preferences, or perceptions formed while listening to musical pieces [40]. Processing music therefore requires a complex analysis of various aspects of the music coming to the ears—an analysis made possible by the development of complex neuronal networks activating numerous parts of the brain, from the brain stem stimulating the higher parts of the nervous system, through the limbic system, to the cerebral cortex, including not only the temporal lobes, but also the frontal, occipital, and parietal lobes of both hemispheres [41]. However, the process of branching neuronal connections, which has already begun in the prenatal period, intensifies significantly after birth, which is reflected in the acquisition of further skills in the newborn.

Due to the high sensitivity to the sound of human voice, already developed in fetal life (e.g., demonstrated by the ability to accurately differentiate the frequency of sounds and their duration), newborns show the most obvious and greatest readiness to perceive and learn any language compared to later stages of their development [42]. This includes the perception of a specific culture’s musical language [2]. Despite their readiness to process music from different cultures, two-day-old infants listen longer to a woman’s voice singing in the style of their native culture than to a foreign culture [43], demonstrating the existence of prenatal musical memory. However, openness to the reception of a variety of musical scales from different cultures is the greatest during the first period of life [44].

Delving into more specific aspects of music processing by infants, functional magnetic resonance imaging (fMRI) studies have demonstrated that tonal, harmonious music activates the upper temporal areas in both cerebral hemispheres [45]. In turn, given the preference of newborns for native musical culture, it is worth mentioning another fMRI research study [46] indicating that 1–3-day-old newborns from Western civilization, when provided with musical stimulation in the form of Western tonal music, showed activation of the primary and secondary auditory cortex. However, such activation was found to be more pronounced in the right hemisphere [46]. Of note, this pattern of cerebral activation driven by musical stimulation is characteristic for adults who are not music professionals [40]. Interestingly, when the same musical fragments were changed to be less tonal, activation in an infant’s right auditory cortex decreased in favor of the dominance of the left superior and middle temporal areas. Further, there was also involvement of the left inferior frontal cortex and structures of the limbic system (left amygdala, left ventral striatum) [46]. This means that newborns are already equipped with the memory of the musical structures of the culture in which they were born. In addition, they develop a process of analysis when they hear changes in a familiar musical system—a process in which the left hemisphere of the brain plays a dominant role. In addition, an emotional response is triggered when these children hear music that is different from what they have heard before, which additionally activates the limbic system. This demonstrates a kind of universal knowledge of the musical grammar of one’s native civilization and a high capacity for the complex processing of music from other cultures. This also provides neural evidence of a preference for familiar melodies, as well as a readiness to learn any musical system from any musical culture early in life. It is worth mentioning that the process of complex processing of complicated sound material, such as music, is just starting in the neonatal period and develops at a rapid pace [3,15].

Interesting data on neonatal brain activity in response to musical stimulation are also provided by the results of EEG studies conducted on the second and third days of the baby’s life. A 2-beat percussion rock accompaniment composed for a snare drum, bass drum, and hi-hat was used as stimulation. When presenting newborns with this accompaniment, it was found that babies have an innate ability to detect beats of rhythm in a musical sequence. Indeed, EEG revealed beat anticipation in the presented rhythmic sequence (amplitude peak at 200 ms before and 600 ms after sound presentation) [47]. These results confirm that infants (as young as a few days old) have the aptitude to temporally process music.

Perhaps the strongest evidence for musical memory and musicality developing as early as in the neonatal period comes from research on maternal voice recognition and on the enormous role of maternal singing that has been shown to impact not only the musical but also the holistic development of the newborn child. As mentioned above, newborns respond almost unerringly to their mother’s tape-recorded voice and distinguish it from the voices of other women [29,30,48]. Moreover, they modify their behavior to activate their mother’s voice instead of another woman’s voice [29]. At the sound of the maternal voice, they calm down, which is manifested by a slower pulse [49], lower mobility [50], and more stable behavior [51]. This calming effect of the maternal voice applies to both her speech and singing [2]. The soothing effect of maternal singing, heard as early as in fetal life, plays a huge role in early cognitive and emotional development [52,53], mainly by regulating tension and creating emotional bonding [2]. Distinguishing between pleasant and unpleasant emotions in the mother’s vocalizations becomes the basis for the child’s later emotional experience of music, which is the central axis of musicality [2]. It also appears that newborns prefer to listen to speech sounds over non-speech sounds and to native language over foreign language, thus indicating native language memory originating in fetal life [54].

It is also worth considering whether music in the neonatal period can support the process of learning—for example, in the case of the sucking reflex. In a study of preterm infants, each moment of pacifier sucking was associated with lullabies sung by women played from recordings, thus conditioning the sucking response. An ascending learning curve was observed; that is, there was a systematic enhancement of the sucking activity during periods of musical stimulation [55]. In addition, music used as reinforcement resulted in improved milk suckling from non-nutritive to nutritive, affecting neonatal well-being [56]. Another study conducted in the NICU (Neonatal Intensive Care Units) showed that the use of music therapy improved neuropsychological development in hospitalized newborns, compared to a control group who received standard care [57].

Summarizing the results of research on the broadly understood musical development in the neonatal period, it can be noted that the child comes into the world with a fully formed sense of hearing. The child demonstrates the ability to differentiate the melodic and temporal aspects of incoming sound stimuli, which is associated with musical and rhythmic hearing. It performs a simple analysis of the changes in music and reacts to them with an emotional response. In music perception, it obviously does not yet reach the same level of complexity and understanding as an adult. However, it has a prenatal musical memory and remembers new music fragments. The baby prefers its mother’s voice, and in its relationship with the mother, it learns to experience music emotionally, as reflected in the development of musicality. Over the course of this musical development, the interaction of genetic factors with environmental influences is also emphasized. Therefore, optimal musical stimulation that does not overload the developing nervous system, based on maternal vocalizations and (to a lesser extent) on musical pieces, is very important in the first month of life. After all, although, initially, an infant’s basic physiological needs and the establishment of a safe relationship with a significant person (e.g., mother) seem most important, musical stimulation—by contributing to the musical development—can significantly help to fulfill these needs [4].

## 4. Infancy

Infant musical development occurs primarily in the context of maternal bonding and maternal singing [2,5,6]. Mothers in all cultures of the world tend to speak more musically to their infants than to adults [2]. This phenomenon is called ‘motherese’ or infant-directed speech. It is characterized by a singing style with higher sound frequencies and an extended range of sounds, extended vowels, and a slow tempo [58,59]. In addition, mothers tend to ask their infants a lot of questions (rising melodic contour of the sentence) even though their children cannot yet answer them linguistically. This sentence suspension at a higher frequency focuses an infant’s attention [60]. It is likely that this style of speaking serves to establish contact with the infant and elicits prelinguistic readiness in the infant.

Remarkably, all over the world, mothers and other caregivers of infants practice singing, adding it into daily activities. They sing while feeding, changing diapers, bathing, playing, or preparing for sleep [61]. Further, although the repertoire has its cultural specificity, lullabies, sing-alongs, and children’s songs can be distinguished everywhere [62]. Compared to infant-directed speech, singing songs does not exhibit such significantly elevated frequencies or a slower tempo. It is far more expressive and emotional than singing without young children present [61]. While speaking to infants is usually three or four semitones higher when compared to speaking to adults, singing to infants is most often only half a tone higher compared to singing to adults. The rhythm of the songs is preserved, but the dynamics are increased, while the timbre of the voice gains a soft tone [61,63]. Repetition in terms of repertoire is also emerging. These distinctive features of speech and singing to infants serve to establish an emotional bond between the child and the caregiver. Higher pitches and slower vocalizations are associated with happiness and emotion [64], and the regularity of rhythms in sing-alongs and children’s songs promotes coordination with movement and the creation of emotional communication between mother and infant [65]. The simplicity in the tonality of maternal singing encourages reciprocation of vocalizations as expressed by the child [66], and familiarity with songs, often developed from the prenatal period, has the effect of strengthening bonds, increasing feelings of security, and bringing a sense of comfort and pleasure [61]. Infants also respond to the emotional context of music. This is because they prefer the happy tone of voice and smile that accompanies the mother when singing or speaking [67]. An interesting study documenting the role of singing in the formation of emotional bonding was conducted in Ireland [68]. During four group sessions, six women participating in birthing school classes were taught very melodic lullabies, unfamiliar to the participants. After several months, the study participants reported that the songs learned during the study were a valuable way to express the deep feelings they felt for their child. They also evoked feelings of calmness, relaxation, and closeness. Moreover, the women felt that the infants recognized these lullabies by responding with movements and vocalizations while listening [68]. In a similar study in Australia, women were taught how to use lullabies to evoke an atmosphere of calmness and safety for their infants. It was found that singing lullabies to babies also affected the emotional and physical well-being of mothers [69]. The benefits of singing lullabies can therefore apply not only to infants but also to their mothers. This conclusion is further supported by the results of a study on the use of music therapy with preterm infants and their parents in the NICU. Indeed, participation in music therapy supported parents by increasing their well-being, confidence, and the quality of interactions with their preterm children [70,71]. Similar results were also obtained by Haslbeck and Bessler [71]. These researchers demonstrated that an appropriately designed Time Together educational program designed to encourage mothers to utilize infant singing resulted in an increased sense of the mother’s own efficacy [71].

The prevalence of singing to infants suggests that humanity is somehow biologically programmed to use singing or ‘motherese’ in its earliest interactions with infants. Around the world, both mothers and fathers and other caregivers use their musicality to connect and bond with their infants, and to establish a kind of reciprocity, as the relationship is bilateral. This is because the child responds to the musical activity of the caregivers with vocalizations and movement. Thus, it can be seen that singing and music are used to communicate with each other in the first year of life [72]. One can go as far as to say that infants ‘teach adults how to perform for them’ [73] p.2. The musical dialogues that emerge become increasingly infused with reciprocity, meaning, and emotion by mimicking and complementing rhythmic pulse, dynamics, melodic contour, or timbre. These sung conversations foster not only emotional bonding, but also emotional adjustment, language learning, and, crucially, musical development [6,73].

Considering benefits of singing to infants, it is worth noting that it has a positive effect on agitation and stress levels. Six-month-old infants showed significant reductions in cortisol levels after listening to their mother sing. Infants became calmer if they were previously upset or more agitated if they initially showed drowsiness [74]. Listening to music leads to a reduction in agitation (manifested as a decrease in cortisol), even in infants who do not exhibit elevated stress levels [75]. It has also been reported that singing is more effective than ‘motherese’ in regulating stress levels in infants [76].

Lowering tension and experiencing well-being by listening to the mother sing, as well as sharing a musical communication with her, serves emotional and social development in a broad sense. Emotional signals conveyed in song by the mother are noticed by the infant, as the infant is born with high auditory sensitivity. Moreover, the child not only learns to pick up emotional signals in other people’s voices, but also responds to them through musical dialogues, gradually acquiring an awareness of emotions and the ability to express them [63]. On the other hand, sensitivity to one’s own and others’ emotions helps with social development. Learning music can also have such benefits. Considering the results of neuroimaging studies, we know that music engages regions responsible not only for experiencing but also for regulating emotions (both limbic and paralimbic systems, including the orbitofrontal cortex) [41]. This means that musical interactions may affect social development by activating specific cortical–subcortical systems [24]. However, compared to passive music listening, active participation in group music activities has been shown to have a stronger effect on brain development, improving communication and the social–emotional development of infants [77,78].

To summarize, a mother’s singing does not only facilitate musical development, but also provides invaluable assistance in bonding and creating secure attachment, bringing emotional benefits to both the infant and its mother [4]. Singing allows us to experience pleasure and joy together. It also offers an effective way to express a variety of emotions, reducing stress levels. It affects psychomotor development and cognitive functions, stimulating the child’s attention, influencing the activation of memory processes and learning. In addition, it plays a role in social development through musical dialogues [2].

What makes musical stimulation, especially the mother’s singing, so beneficial to infants both emotionally and cognitively? First, music perception activates numerous areas of the brain. The right temporal lobe is dominant for sound pitch perception [40,79]. Further, during pitch and melody perception, also the cerebellum is activated, particularly its left hemisphere, which has connections with the right cerebral hemisphere [80]. The left temporal lobe [40], the prefrontal motor cortex, and the cerebellum [81] are also involved in rhythm perception, which additionally activates the basal nuclei and the cingulate cortex [80]. However, both the basal nuclei and cingulate cortex may be involved not only in rhythm processing but also in alertness. The mesencephalon also takes part in processing the temporal aspects of rhythmic structures, probably due to the excitatory role of rhythm [80]. Motor responses to the music further activate the motor and sensory areas of the cerebral cortex. Numerous additional areas associated with the entire network of associations; expectations, memory, or meanings are engaged as more and more auditory experiences are acquired [82]. This includes the orbitofrontal cortex, the activity of which is observed in adults when listening to their preferred music [83].

Therefore, it can be assumed that, since listening to music activates a significant part of the brain, the processes activated during music perception can be generalized and transferred to functions unrelated to music, thus yielding benefits for other cognitive processes [84,85]. Music can promote learning through the aforementioned fact of attracting an infant’s attention. Listening to music, especially music associated with the sensation of pleasure, activates interconnected subcortical and cortical neuronal networks that include the ventral striatum, nucleus accumbens, amygdala, insula, hippocampus, hypothalamus, ventral tegmental area, anterior cingulate gyrus, orbitofrontal cortex, and ventral medial prefrontal cortex [83,86,87,88].

The mechanism(s) accounting for music’s positive effects on infant cognitive and emotional functioning can also be sought at the neurotransmitter level. For example, the ventral tegmental area that produces dopamine has direct projections to the sinusoidal area, the amygdala, the hippocampus, the anterior cingulate cortex, and the prefrontal cortex [89,90]. It has been suggested that the responses of the ventral tegmental area and the nucleus accumbens are associated with the suppression of aversive stimuli and pain [83], whereas the sinusoidal site and hypothalamus mediate agitation. By acting together, they form a dopaminergic system that is extremely important for arousal, experiencing emotions, and activating the reward system, as well as for motivation, memory, attention, and executive functions [89]. Studies suggest that increased dopamine levels enhance alertness, information processing speed, attention, and memory [91]. Thus, the pleasure experienced by the infant when exposed to music, especially the mother’s singing, is associated with the stimulation of the dopaminergic system involved in the attracting of attention, thereby improving cognitive functioning. This approach seems consistent with the arousal and mood hypothesis formulated by Thompson and coworkers [92], who posit that any stimulation that induces positive emotions and increases arousal (e.g., appropriately selected music) leads to improved performance in cognitive tasks [92]. In addition, listening to music, as already mentioned, reduces stress (by lowering the cortisol levels), thus providing a greater opportunity to experience pleasurable emotions.

It is worth emphasizing that, every day, the infant encounters new stimulation, including sound. Some of the stimuli will be heard repeatedly, which will contribute to its memorization. According to the concept of Elkhonon Goldberg [93], the perception of completely new information involves mainly the right cerebral hemisphere, while processing of already known information takes place with greater participation of the left hemisphere. Thus, over the course of learning, there is a transfer of activity from the right to the left hemisphere. During infant development, this will also be reflected in the ability to learn language—from the prosodic aspects of speech and singing to understanding the meanings of words and language expression. In addition, processing music, which intrinsically evokes specific emotional states, involves the right hemisphere to a greater extent (for review, see [94]), analogous to the understanding of the mother’s emotional prosody [95]. Listening to music or maternal singing may therefore stimulate the infant’s right hemisphere to a greater extent, contributing to the infant’s increased levels of norepinephrine responsible for the exploratory behaviors characteristic of the first learning stages [93]. Due to the fact that listening to music, especially if accompanied by a text (songs, vocal–instrumental music), activates bilaterally significantly more neuronal networks than listening to verbal material alone [96], it can be concluded that musical stimulation is superior to language-only stimulation in child development, especially in terms of developing musical aptitude.

Therefore, it seems reasonable to discuss how particular musical aptitude is formed in the first year of life. In terms of melodic hearing, pitch perception has been described as surprisingly good, even shortly after birth [97]. After several repetitions of an instrumental piece containing five to ten notes, infants are able to notice a slight change in pitch in the form of a half tone in a single note of a given melody, even when the entire melody is shifted to a different key. In the perception of the piece, however, it is the melodic contour that seems to be the most significant feature. Nevertheless, infants can sometimes detect interval changes even when the melodic contour remains unchanged [98]. They show a better ability than adults to perceive small differences in pitch [99], which, as already mentioned, is associated with a high potential to perceive tones from different cultures. For example, adults can detect a slight change in pitch, even less than half a tone, in music from the native major–minor scale. However, they cannot perceive the same change in culturally foreign music because their implicit knowledge of the major–minor scale structure interferes with the perception of melodies with a different organization [100]. In contrast, 9-month-old infants distinguish between changes in the two types of music, because they are not yet as strongly aurally based in the structure of the major–minor scale [97]. At 3 months of age, babies perceive octaves as equivalent sounds [101]. In general, infants prefer consonant intervals (pleasant and congruent-sounding distances between sounds), associated with feelings of harmony, peace, warmth, and safety, rather than dissonant intervals (discordant-sounding distances between sounds), giving the impression of tension or lack of harmony [35]. Evidence for this type of preference is provided by the results of an experiment, in which 4-month-old infants showed dissatisfaction reactions when single lowered sounds were added to a melody with harmonic simple accompaniment, evoking a sense of dissonance [102]. Furthermore, 6-month-olds are more likely to listen to consonant intervals such as the fifth and octave than to the tritone, which represents a dissonant distance between sounds [103]. In musical passages, in addition to consonant intervals, infants prefer harmonious consonances, small distances between notes, a diatonic scale, and tend to focus on the contour of the melodic line [97].

In terms of rhythmic aptitude, infants present high rhythmic sensitivity, recognizing subtle changes better than adults. They are able to discern differences in rhythmic grouping, even in the context of competing tempo changes [104]. They focus on the relative rather than the absolute duration of the sound [97]. Seven-month-old infants can categorize the rhythm according to the given meter [105]. Infants detect changes in duple rather than triple meter more accurately, which is explained by the higher frequency of duple meter in children’s songs and its greater ease [106]. Moreover, how do they deal with compound rhythms? It turns out that adults from Western culture can detect changes in the rhythms of Balkan music if it is in duple or triple meter, but they cannot cope with complex rhythms. By comparison, 6-month-old infants can detect changes in both simple and complex rhythms perfectly. However, what happens after another six months of their lives? Twelve-month-old children process rhythms already, similarly to adults; that is, they are more sensitive to simple rhythms, while making mistakes in complex rhythms. This means that the enculturation process is already taking place at this stage of development, although these children still show greater flexibility in learning foreign musical material compared to adults [107].

In the context of rhythmic capacity, it is of interest whether rhythmic processing abilities play a role in native language acquisition. It is known that particular languages of the world differ in rhythmic aspects (syllable length, accents, etc.), while the other ones show compatibility in this respect [108]. Based on an interesting study [108], it was found that 5-month-old American infants discriminated pairs of languages from different rhythmic classes (Italian vs. Japanese, British English vs. Japanese), but they did not discriminate two languages within a foreign rhythmic class (Italian vs. Spanish). Similarly, in another study [109], infants were able to discriminate between Japanese and Dutch. These results confirm the previously discussed relationship concerning the high processing capacity of rhythm in infants—in this case, the rhythm of language. Moreover, for language discrimination, processing of the rhythm of the heard speech seems to be more important than melodic elements (since there is no distinction between similar languages in terms of rhythmic aspects) [108]. In addition, another experiment within the discussed research showed that language discrimination occurs better when comparisons involve the native language and a language from a foreign rhythmic class [108]. This implies that at 5 months of age, enculturation in the native language is already strong, whereas rhythmic processing abilities facilitate language acquisition [108].

Musical memory develops rapidly during infancy. The abilities described earlier to detect differences in pitch or rhythm require memorization of presented musical passages, and infants excel at this. However, can they already memorize entire pieces of music? In an experiment described by Sandra Trehub [110], infants listened to a selected Mozart sonata and a folk tune for two weeks. As expected, they were able to respond to the melodic–rhythmic change introduced at the next repetition, proving the existence of musical memory. In addition, mothers of infants also report that children in this period quickly learn new songs and recognize them by responding with smiles, vocalizations, or movement [6,111]. For a piece of music to be memorized, the infant’s attention must first be focused on the sounds presented. It has been observed that in 6–9-month-old infants, baby-directed singing is significantly more effective for attracting attention than recorded music [112]. In addition, in comparison to episodes of maternal speech, maternal singing augmented with visual elements (smile, gaze, etc.) was associated with a better ability to sustain attention (as revealed by concentration times) [65]. Of note, in infants, focusing attention as well as an increase in alertness have been shown to be associated with a significant reduction in movement [113].

In infancy, music-related movements and playing with objects that make sounds (such as rattles) constitute preparation for possible future learning how to play a certain instrument. After the 6th/7th month of age, spontaneous movements to music become more frequent [114]. In addition, although this early movement response to music (manifested by, e.g., rocking, moving the head and other body parts, stomping, kicking, clapping, and, in the case of a rattle, shaking it or throwing it [3,97] is very rhythmic in nature, at this stage of development, the movement itself is not coordinated with the musical rhythm and pulse.

In addition to music-related movements, singing is also a natural musical activity. As mentioned earlier, it occurs through the contact with the ambient sounds of the native culture [5]. Both the singing style of speaking to infants and the mother’s singing encourage the child to imitate and join in the musical dialogues. The earliest type of vocal behavior is crying, which includes vocalizations of varying pitch, intensity, rhythmic aspects, and phrasing [115]. At two months of age, the infant is cooing, which is associated with a predominance of velar consonants (kgh), although vowel-like sounds also occur, and two to three months later, the infant is babbling. Babbling incorporates typically musical qualities such as pitch and rhythmic patterns (repeating one syllable, such as bababa, then different syllables, such as badag). It is important to stress that the extent, quality, and timing of the emergence of babbling depend on the amount of time the mother spends singing. The more mother sings, the greater the likelihood of earlier onset of babbling [5]. In addition, prenatal and neonatal musical experiences are also important: if they are rich, vocalizations and musical babbling appear more quickly, in greater quantity, and have greater musical value in 2–8-month-old infants than vocalizations in children who do not receive musical stimulation in fetal and neonatal life [116]. By 3–4 months of age, the infant can imitate the expressive prosodic contours of the mother’s speech [117], and between 4 and 6 months, voice playing appears [72]. The first signs of singing can be noticed in the second six months of life. At first, they take the form of the articulation of syllables with extended vowels in a fixed key, then improvisation on syllables with a characteristic glissando figure, to resemble songs with sequential organization of sounds around 1–1.5 years of age [118]. By the age of 12 months, the child is already immersed in the sounds of its culture, which manifests itself, among others, in using vocalization with sounds characteristic for the native language—infants in France chatter ‘in French’; in Russia, ‘in Russian’; and in Japan, ‘in Japanese’ [119]. Thus, the first year of life is a period of increasing diversity of vocal activity: from the first vocalizations expressing experienced emotions such as discomfort, stress, or well-being, through the incorporation of musical properties into one’s vocal productions (between 2 and 4 months), the development of control over one’s voice (4–7 months), to melodic vocalizations with melodic contours and language-specific sounds [5].

## 5. Theories of Early Musical Development

There are several theoretical directions in the music psychology literature that emphasize the role of early auditory experiences in musical development [120]. The classic approach is presented by Helmut Moog [121], who observed children’s musical development from infancy to adolescence in the context of holistic psychosocial conditions. According to him, infants respond to music with movement. This is followed by an interest in locating the source of the sound and a self-expression in response to the sound, taking the form of the first imitative vocalizations. Music also begins to have a relaxing function. In the postnatal period, spontaneous motor activity associated with vocal activity is more often externalized. The quality of singing improves, and movement to music often occurs in the form of social games. Gradually, attention to the music and coordination of movement to the music improves. The need for vocal creativity also becomes apparent. The child slowly grows into the native musical culture, but, according to Moog, enculturative influences before the age of 3 are still difficult to identify [121].

Edwin Gordon [44] presents the approach that innate factors are the root of musical aptitude. In addition to genetic factors, the musical environment, affecting the mother during pregnancy and the fetus’s responsiveness to music, plays a large role. According to Gordon, at birth, a person presents with the highest potential for musical aptitude, which declines steadily from that point on. The presence of musical stimulation can slow or inhibit this process. However, the effect of the environment only matters until the age of 9, when the musical aptitude stabilizes. Gordon uses the concept of audiation, which he defines as musical thinking, hearing, and understanding music. He also distinguishes two types of contact with music: musical direction and education. In contrast to musical education (associated with schooling), Gordon describes musical direction as stimulating the child with music as early and as comprehensively as possible, preferably from the prenatal period, and to undertake musical play that engages the child in what they hear. The greater the variety of music used, the better. The goal of direction is to expand musical experience and inhibit the loss of innate aptitude, not to acquire specific musical skills [44].

Another approach, rooted in the cognitive psychology of music, is represented by John Sloboda [122]. He considers music in language categories. The interaction of self-activity with genetic factors and environmental influences is responsible for musical development. This author divides musical development into two stages: enculturation and adequate training, most often associated with institutional education. Enculturation as a process of growing into the culture of a given society includes the everyday experience of cultural products, including music. Learning music is similar to acquiring language—it is unconscious and often occurs through imitation. Analogous to language, music-receptive abilities emerge more quickly than expressive ones. During this period, which lasts until roughly the age of 10, there is a lack of self-consciousness about the development of musical skills, and their acquisition is somewhat automatic due to the influence of the environment (mainly the home). Thus, musical development occurs in the context of cognitive development and experiential learning processes in the musical culture in which the child grows. During the enculturation period, musical awareness begins to develop, enabling them to perceive specific relationships between different sounds; this process can be noticed already around the 5th month of age. According to Sloboda, conscious musical behaviors cannot be observed before the age of 6 months; in fact, in some cases, they may not be present until the age of 1 year. Although intensive training can help with the development of pitch imitation, this skill is more related to the ability to copy the intonational contour of speech. Sloboda argues that during the first year of life, infants only distinguish between musical and non-musical sounds, as demonstrated by their focus of attention, vocalizations, or motor responses. The emergence of spontaneous singing around 18 months of age is considered a significant milestone in musical development. The child begins to use pitch-stable sounds, initially within seconds and thirds, and later also quarters and fifths. In years 2 and 3, songs show an increasing level of organization, as manifested by relatively tonally fixed phrases, consciously used repetitions, or a wandering tonal center. This indicates that the music has begun to be treated as a constellation of interval rhythmic patterns associated with a specific set of intervals. In the postnatal period, the child incorporates snippets of familiar songs into his or her singing. Next, the awareness of the rhythmic and melodic patterns characteristic of the native culture comes. Gradually, the intonation accuracy of singing increases to the point where children around the age of 5 can correctly sing songs from their cultural background. Increasing the child’s self-awareness of performance has the effect of gradually displacing spontaneous singing in favor of precise copying of existing songs or even imitating specific performances of them. A 5-year-old child can demonstrate conscious concentration on key and rhythm structure as well as demonstrating a proper coordination of movement to music [120].

Along the same line, also Maria Manturzewska and Barbara Kamińska [121] posit that musical development is a result of genetic and innate factors that determine the range, upper limit, and growth dynamics of potential predispositions. The authors also point to the importance of environmental factors. Specifically, the direct availability of examples of musical behavior from the earliest childhood, the inclusion of music in the process of natural communication with the immediate environment, and systematic practice of basic musical skills (singing or playing an instrument under the guidance of a competent person) seem essential for the realization of musical aptitude. Hence, development largely depends on a musical dialogue with the environment. The same researchers also emphasize the importance of critical periods in the formation of particular skills. In prenatal life, the first sensorimotor responses to music appear in the form of motor responses. During infancy, sensory–emotional sensitivity to music increases, and reactive–emotional musical activity becomes evident. Focusing on the human voice and singing inspires a child to manipulate their voice and to vocalize. Before the age of one, the child distinguishes between singing and speech, and starts singing songs, although the melodies that they bear no resemblance to patterns. Musical memory becomes apparent, as evidenced by the child’s recognition of different sounds as well as frequently heard melodies. Spontaneous musical activity, both vocal and motor, becomes more important in the postnatal period. At this stage, large individual differences can already be observed in the level of musical development in terms of musical memory, attention to music, or precision in reproducing melodies [121]. Although each of the approaches presented above emphasizes a slightly different dimension of early musical development, all point to the role of early musical stimulation in the development of musical aptitude.

## 6. Conclusions

Early prenatal listening experiences as well as musical stimulation and the mother’s voice have a strong effect on the development of musical aptitude. Musical development begins very early in the prenatal period, taking place in the context of unconscious exposure to the influences of native musical culture. The development of individual musical aptitude is facilitated by both innate factors and the influence of the environment, especially the family. The child’s own activity is also very important. Mother’s singing and musical dialogues with a child play a huge role, bringing many other benefits that may extend far beyond musical development itself. This is because they facilitate bonding and contribute to the development of emotional, social, and cognitive functioning. The positive effects of musical stimulation on children’s development, including their cognitive and emotional functioning, have neural determinants. Not only fluency in music perception but also spontaneous movement to music and vocalizations represent manifestations of early musical development. Furthermore, listening to music and actively playing music bring a lot of beauty and joy to everyday life, adding to it an aesthetic value essentially from early childhood.

Despite all the evidence supporting the notion that early musical stimulation has a beneficial effect on child development, the psychology of music is still in infancy, especially when the impact of early music stimulation on the development of other skills is considered. Further, it would be interesting to see if the benefits of early auditory experiences remain relatively stable over time and/or have a potential impact on aging and cognitive reserve. Moreover, longitudinal studies using brain neuroimaging are still needed to comprehensively evaluate the contribution of perinatal musical stimulation, including mother’s singing, to the development of cognitive, emotional, and social skills in both infants and preschool children. Moreover, in the context of brain neuroplasticity, future research is warranted to see if the beneficial results of early musical stimulation can also emerge when the musical stimulation is provided only later in life, e.g., during schooling.
